# Investigation on the Mechanical Recycling of Carbon Fiber-Reinforced Polymers by Peripheral Down-Milling

**DOI:** 10.3390/polym15040854

**Published:** 2023-02-09

**Authors:** Massimo Durante, Luca Boccarusso, Dario De Fazio, Antonio Formisano, Antonio Langella

**Affiliations:** Department of Chemical, Materials and Production Engineering, University of Naples Federico II, Piazzale Tecchio 80, 80125 Naples, Italy

**Keywords:** recycling, CFRP, peripheral milling, specific cutting energy, mechanical testing

## Abstract

Carbon fiber-reinforced plastics (CFRPs) are composite materials that play a significant role in the growth of many industrial fields where high performance and lightness of the structures are required. At the same time, the management at the end of their life has required the development of more and more sustainable and efficient recycling solutions. Considering this, the present research work aims to investigate a mechanical recycling method and the cutting strategies able to machine CFRP components in their entirety, using a common milling machine in a job shop scheme, making a shorter supply chain, and leading to economic and environmental benefits. In detail, laminates obtained by unidirectional carbon fiber prepregs were worked through the peripheral down-milling process, by varying the spindle speed and the feed rate. The recording of the cutting forces enabled the evaluation of features such as the cutting power and the specific cutting energy. Moreover, the chips from the milling process were classified as a function of their dimensions. Finally, specimens made of chips and epoxy resin were characterized under bending conditions, to evaluate the effectiveness of using the chips from CFRP peripheral milling as the polymer’s reinforcement and, in addition, to appreciate the goodness of this recycling strategy.

## 1. Introduction

In recent decades, the development of CFRPs has attracted significant attention; consider that the demand for this class of materials has tripled from 2010 to 2020 and could surpass 190 kt by 2050 [1,2]. This success is a consequence of their excellent mechanical properties, combined with lightweight, due to the superior strength-to-weight ratio of the carbon fibers (CFs) that act as load-carrying elements in the polymer matrix. Components made of CFRP can be used for applications in different fields, such as aerospace, automotive, construction, and energy [3], often replacing the most common metals and alloys [4]. Inevitably, the high demand introduces disadvantages concerning the dramatical increase in CF and CFRP waste in the coming years; consequently, concerns about the management of these products at the end of their service life have been rising [5]. Consider that, regarding only global CFRP waste, there is an annual growth perspective of 20 kt by 2025 [6].

Researchers and industry are experiencing the need to develop industrial production systems to improve the efficiency of the resource utilization and to reduce resource consumption and environmental pollution [7], due to their large-scale implications for global sustainable development [8]. Then, they are working in line with the principles of the circular economy since there is a strong orientation toward reusing, remanufacturing, and recycling products at their end of their life. Just think that the European directive 2000/53/EC points toward the recovery, reuse, and recycling of almost all the total vehicle components at the end of their life cycle [9], and similar decisions have been made in the aerospace industry [10]. Concerning CFRPs, there are three main strategies to manage waste: disposal (landfilling); energy recovery (incineration); and recycling (e.g., mechanical, chemical, and thermal methods). Landfilling is the most common and cheapest method of disposal. In general, the disposal of waste is carried out in sanitary landfills; they are constructed by lining an excavation with gravel, bituminous concrete and polyethene sheet, to create a barrier between the waste and the surrounding environment. A drainage system is also incorporated; in so doing, a watertight collection system for the leachate, generated during the operation of the landfill, is created [11]. However, US and EU authorities have put restrictive legislation in place on landfilling organic materials (such as CFRP) [12,13], because of related problems such as toxins, leachate, and greenhouse gasses. With incineration, it is possible to recover some energy from waste products. In more detail, it represents a thermal waste treatment process that converts waste material into flue gas, ash, and heat; in so doing, it is possible to release embodied energy and reduce waste volumes [11]. Despite this, it releases large amounts of pollutants into the environment; therefore, it is not considered a sustainable solution for managing waste in the long-term [14]. Finally, the recycling of CF-based products is an emerging research area that will be able to cover, soon, a significant role in a circular economy approach for this material’s family. They represent energy-intensive materials and, consequently, ideal possible candidates for recycling. Nevertheless, it is worth highlighting that there are some difficulties to recycle them. Regarding CFRPs, they widely use cross-linked thermoset polymers for their matrices that cannot be remelted or remolded. Moreover, CFRPs rarely are made of only CFs and a matrix but frequently represent complex multiphase waste types because the base constituents are combined with other reinforcement types such as, for example, glass and aramid fibers, core materials, paints, and metallic inserts. In addition, the waste products can show variability, because there is no standard composition for CFRPs and, finally, the identification of different compositions is technically challenging, with problems of collection and separation from other waste types [15].

Recycling methods can be evaluated in terms of some important issues such as the low emission of greenhouse gasses [16], the energy usage [17], and the properties of recycled materials [18], and CFRP recycling can be categorized into three broad methods, i.e., thermal, chemical, and mechanical, as a function of the energy source to separate the CFs from the matrix. Thermal recycling methods use high temperatures (pyrolysis) and fluids (fluidized bed recycling) to break down the matrix and recover the fibers. Chemical (and electrochemical) methods of CFRP recycling use both supercritical and subcritical solvents, often operating at ambient pressures. The electrochemical recycling methods decompose the CFRP components using high electrical currents, whereas catalytic depolymerization allows to achieve a high degree of clean, residue-free CFs. Finally, the mechanical recycling represents the most promising way to recover CFs from CFRP components since it does not require the use of a high temperature or chemical substances to decompose the polymeric matrix. These aspects lead to a further advantage of this method because the recycling fibers are not subjected to thermal or chemical alterations; therefore, it can be applied without any limitation to the most used industrial fiber’s typologies. Moreover, it can be considered as a valid alternative to the other recycling methodologies because it is characterized by a technology readiness level (TRL) index in a range of 6–7, compared to 3–4 and 4–8 of chemical and thermal processes, respectively [19]. Finally, other features that characterize mechanic recycling and make it very attractive are the rapid industrial scalability and the environmental and economic sustainability in terms of the specific energy demand; it is estimated in a range of 0.1–4.6 MJ/kg, in comparison with about 38 and 40 MJ/kg for chemical [20] and thermal [21] recycling processes, respectively.

Mechanical recycling is mainly based on the reduction of CFRPs into small pieces through shredding and milling/grinding [22]. This method involves the shredding of the components in pieces of 50–100 mm, especially for the removal of metal inserts and to facilitate transport. Then, the chunks are ground into fragments of 50 μm–10 mm using a cutting or hammer mill. Finally, the fragments are separated in their contents and size, through cyclone machines and sieves [15]. Many research works have been focused on the mechanical recycling of composite materials. For example, Palmer et al. [23] recycled CFRP materials aiming to use the recovered CFs in place of glass fibers (GF) in a sheet molding compound (SMC). The composite materials were recycled by using a rotating hammer milling process and the recovered CFs were selected by dimension using a sieving apparatus. Then, recycled CFs with a dimension between 0.5 and 10 mm in conjunction with coarse fibers (longer than 10 mm) were used to produce SMC materials. At the end of the experimental campaign, they pointed out that this category of recycled material possesses mechanical properties comparable with the same materials reinforced with virgin GFs. A shredding recycling process was used in [24] to recycle GFs that derive from wind turbine blade structures. As well as in [23], the research group sieved the recycled fibers to collect them by dimension, then produced different sample typologies as a function of the fiber weight fraction. At the end of the experimental campaign, they observed a not expected reduction in the mechanical properties that varied from almost 40% to around 70%, depending on the recycled material amount. A better insight on the failure surface of the samples revealed the presence of an old matrix and the occurrence of debonding and a pull-out mechanism that, altogether, affected the overall mechanical properties of the recycled composite material. A milling process was employed in a study on the mechanical properties of recycled composite materials reinforced with glass and carbon machined fibers [25]. The work investigated the effect of the fiber’s weight fraction, the matrix degassing, and the addition of an interface coupling agent on the composite’s mechanical performance. At the end of the experimental campaign, the authors observed that the best mechanical properties were reached when recycled CFs were used in conjunction with a coupling agent and the degassing process was carried out. This category of recycled composite materials presented an improvement in the tensile strength, the elastic modulus, and the toughness of almost 12, 19, and 27%, respectively, if compared to the pure epoxy resin.

Then, at the end of a brief review on the actual state of the mechanical recycling technique, it is possible to assert that it could be considered as a practical technique to manage the increasingly large volumes of CF-based waste since it can guarantee the lowering of the emission of greenhouse gasses and energy usage, as well as having characteristics of fast processing and facile scalability; this strategy can be particularly useful for the composites made of a thermoset matrix, for which the recycling process is more complex due to their heterogeneous nature and difficulty in liberating the fibers. In addition, the use of CFRP waste is becoming more frequent as a reinforcement in different cases such as, for example, epoxy composites [26] or epoxy foams [27]. For both the cited cases, the recycled CFRP has demonstrated excellent reinforcing ability for the corresponding materials investigated.

This paper puts itself in the vein of the exploration of sustainable and efficient recycling solutions, an issue of the utmost importance in the mechanical manufacturing industry [28], including rough milling operations [29]; it reports novel features on the employment of peripheral milling for the mechanical recycling of CFRP components. Peripheral milling is generally used for the contouring and finishing of composite components by means of small diameter tools, coated with abrasion-resistant fiber coatings. Instead, in this study, it is used to reduce the components into chips, to recycle them through inexpensive and high diameter tools. Furthermore, the values of the cutting parameters typical of roughing were adopted, to remove high volumes of material and promote damage propagation, in particular delamination; in addition, low spindle speed values were adopted, to preserve the cost-effectiveness of the strategy and to enable the use of low-performance machines. In more detail, the work considered the peripheral down-milling process for the recycling of laminates obtained by unidirectional CF prepregs, for different spindle speed and feed rate values. The cutting forces were monitored, and the cutting power and the specific cutting energy were evaluated; concerning this, the monitoring of the cutting forces can represent an effective tool to collect information on the machining damage mechanisms [30] and to develop energy consumption models [17]. Finally, the relationship between the cutting strategy and the chip dimensions was found and specimens of composite materials made of epoxy resin filled with chips from the peripheral milling were manufactured and tested at bending, to evaluate the capability of the product of the recycling to act as an efficient reinforcement for epoxy resins.

## 2. Materials and Methods

The CFRP laminates considered for mechanical recycling were made of unidirectional CF prepregs (epoxy system EP137-CR509/176, Gurit, Wattwil, Switzerland) in an epoxy matrix (series SX10, Mates Italiana, Milano, Italy). In detail, the layers had a thickness of about 0.2 mm, and were used in a number of 20 following a [0/90]_s_ stacking sequence; consequently, the laminates had a thickness of *t* = 4 mm. They were manufactured with a combination of hand lay-up and compression molding techniques. Each layer was manually stacked up on a mold plate; then, at the end of the stratification phase, the uncured material was placed in a hydraulic press (Carver, Wabash, USA), with a temperature of 130 °C and under a pressure of 8 bars, and was left to cure under these conditions for 8 h. At the end of the polymerization phase, the cured composite laminates had a fiber content of approximately 60% in volume and their density was equal to *δ* = 1.4 g/cm^3^. They were cut into square pieces, 200 mm × 200 mm in size, for the carrying out of the milling tests.

The tools used were HSS-PM three flute end mills (series 14805, UOP S.p.A., Brescia, Italy), 20 mm in diameter, with a length of cut of 45 mm, total length of 110 mm, and helix angle of 40°. They are cost-effective tools for shouldering applications on non-ferrous materials and light alloys, made with powder metal X-85 (Co 8.5%) and titanium- and aluminum-based coating. As anticipated above, the choice not to use high-performance cutting tools was made to preserve the cost-effectiveness of the recycling strategy and encourage the occurrence of failures and delaminations. A 5-axis vertical machining center (C.B. Ferrari, Varese, Italy), equipped with a CNC control system, was used to conduct peripheral down-milling experiments; this cutting strategy was preferred to up-milling in light of the energy-saving considerations resulting from preliminary tests and briefly reported in the Discussion section.

Different cutting strategies were considered, by varying the feed rate, *f*, and the spindle speed, *s*; for all the cases, a depth of cut *d* = 3 mm was considered. The first three strategies were *s* = 500 rpm and *f* = 500 mm/min, *s* = 800 rpm and *f* = 800 mm/min, and *s* = 1000 rpm and *f* = 1000 mm/min; they allowed to investigate any trends depending on the cutting speed parameters under the same feed per tooth *f_Z_* = 0.33 mm/tooth. The fourth strategy was *s* = 1000 rpm and *f* = 6000 mm/min; this high feed rate value, typical of rough milling operations, allowed to increase the material removal rate, *MRR*, and to highlight the influence of *f_Z_* (for this strategy, equal to 2.00 mm/tooth).

**Figure 1:**
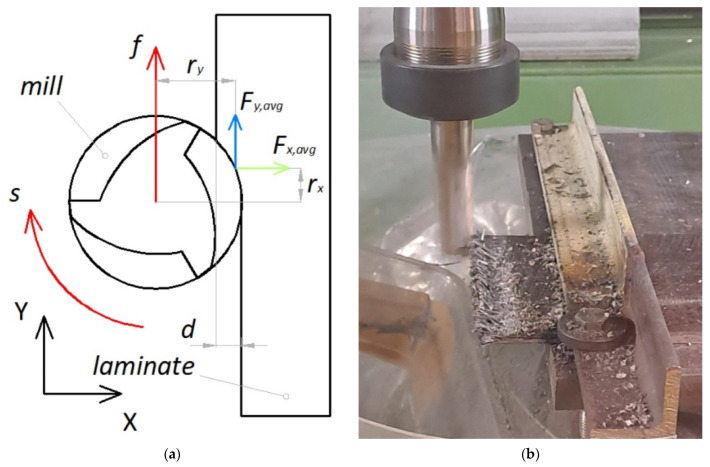
(**a**) Schematization and (**b**) example of the peripheral milling process.

Figure 1 reports a schematization (Figure 1a) and an example of the peripheral down-milling process (Figure 1b). The square specimens were constrained on a side by clamps on the top of a three-axis piezoelectric transducer (type 9257A, Kistler, Winterthur, Switzerland), in turn, fixed on the CNC table; in so doing, the cutting forces (*F_x_* and *F_y_*, see the reference system reported in Figure 1a) were measured. The dynamometer signal was collected and amplified by a multi-channel amplifier and transferred to a National Instruments data acquisition card. The signal was continuously sampled at a frequency of 20 kHz.

From the cutting forces, the cutting power, *P*, and the specific cutting energy, *E_SP_*, were evaluated, to develop energy and environmental considerations [31]. In line with the model presented in [17], *P* was measured as the sum of two contributions (*P_rot_* and *P_trans_*) tied to the two types of motion, i.e., the rotational and the axis feed motion, depending on *s* and *f*. In detail, the mean values of the forces (*F_x,avg_* and *F_y,avg_*) were considered at the center of the arc of contact; *F_y,avg_* and *f* contributed to *P_trans_*, while s, *F_x,avg_*, *F_y,avg_* and the two corresponding radii (*r_x_* and *r_y_*) contributed to *P_rot_*, following Equation (1):

*P* [W] = *P_rot_* + *P_trans_* = [(*F_x,avg_* × *r_x_* + *F_y,avg_* × *r_y_*) × 2π*s* + *F_y,avg_* × *f*]/60000. (1)

All the features of Equation (1) are represented in Figure 1a. Then, the specific cutting energy, *E_SP_*, was measured by

*E_SP_* [MJ/kg] = *P*/(*MRR* × *δ*), (2)

where *MRR* in Equation (2) is equal to

*MRR* [mm^3^/s] = (*f* × *t* × *d*)/60. (3)

**Figure 2:**
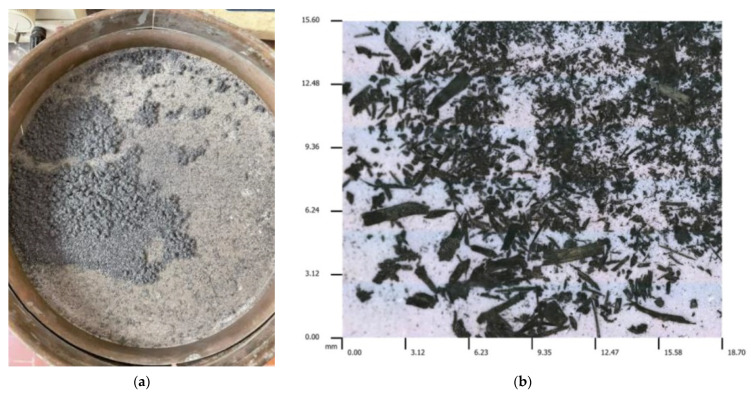
(**a**) Separation by sieving and (**b**) micrography of the chips.

Moreover, the chips obtained by peripheral milling were collected and then separated by sieving. In detail, this operation was conducted using a shaking table assisted by an air flow, aiming to achieve a satisfying separation of fine and coarse chips from the powder of the material to recycle. This separation was carried out with the purpose to distinguish different categories of recycled materials; in fact, the chips were classified into six ranges, depending on their dimension, *d_c_*: *d_c_* > 3 mm; 2 mm < *d_c_* ≤ 3 mm; 1 mm < *d_c_* ≤ 2 mm; 0.3 mm < *d_c_* ≤ 1 mm; 0.1 mm < *d_c_* ≤ 0.3 mm; and *d_c_* ≤ 0.1 mm. Figure 2 reports the operation of sieving (see Figure 2a) and a micrography of the chips (see Figure 2b).

**Figure 3:**
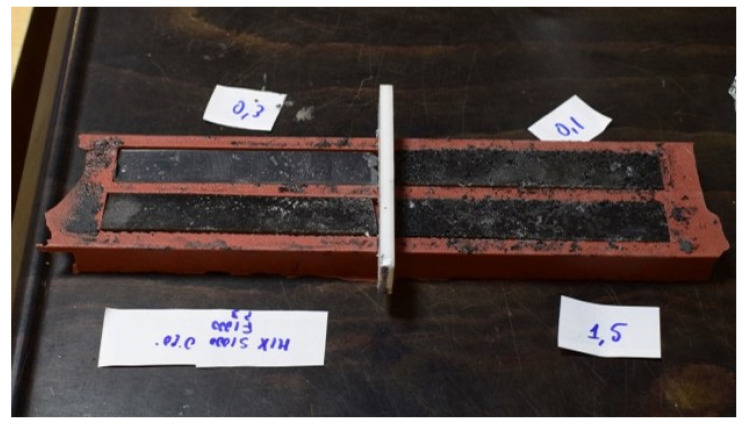
Manufacture of the chips/epoxy composite specimens.

Finally, specimens for bending tests (20 mm × 150 mm and 3 mm in thickness) were manufactured by impregnating the chips with an epoxy resin system (in the weight ratio 1:3, to guarantee a recycled material weight percentage of 25%); specimens of only resin were manufactured too. The resin system considered, with a density of 1.15 g/cm^3^, is widely used as a matrix for composites based on glass, aramid, polyethene, and carbon fibers [32]. The manufacture of the specimens and the execution of the tests were made in compliance with the ASTM D790m standard. Figure 3 reports the manufacture of the specimens by impregnating the resin with recycled material.

The composite specimens were manufactured by manual impregnation and then placed in a mold and left to cure at room temperature for 24 h. Particular attention was paid during this production process because of the possible formation of porosity during the matrix preparation, the development of recycled fibers’ agglomeration that leads to the formation of unimpregnated zones and the formation of voids caused by the random orientation of the recovered fibers. A first matrix degassing was carried out to avoid the formation of premature porosity into the resin bath; then, particular attention was paid to the composite production phase where the recycled reinforcement was gradually added to the resin bath, permitting a good impregnation, and then reducing the possibility of porosity formation.

The campaign of bending tests was designed for an evaluation of the mechanical properties of the chips-based composite material, compared to the ones of only resin, to appreciate the goodness of the chips as a resin reinforcement. In so doing, it is possible to better understand the residual properties of the recovered fibers. Three-point bending tests were carried out using a universal testing machine, Alliance RT/50, equipped with a 1 kN piezoelectric load cell. The tests were performed in a number of 5 specimens for each sample typology. The loading nose was 10 mm in diameter, while a span-to-thickness ratio was fixed to 32:1 to reduce to a minimum any shear effect on the bending behavior of the tested specimens.

## 3. Results

**Figure 4:**
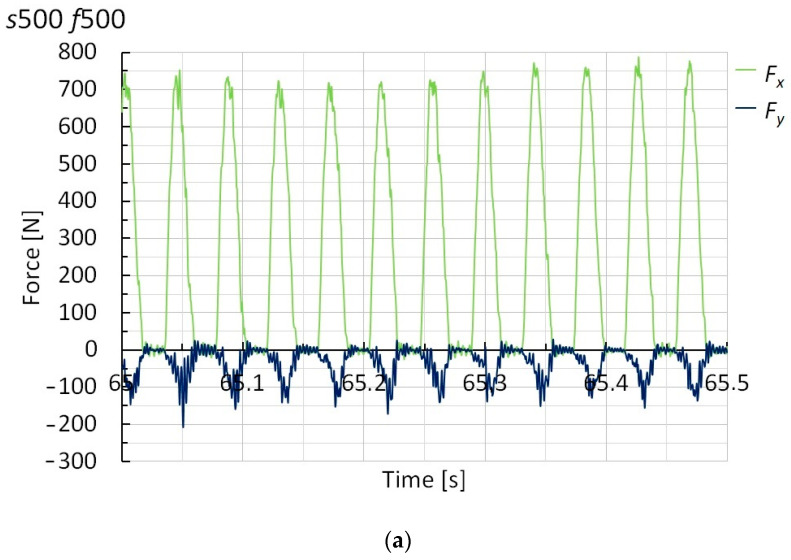

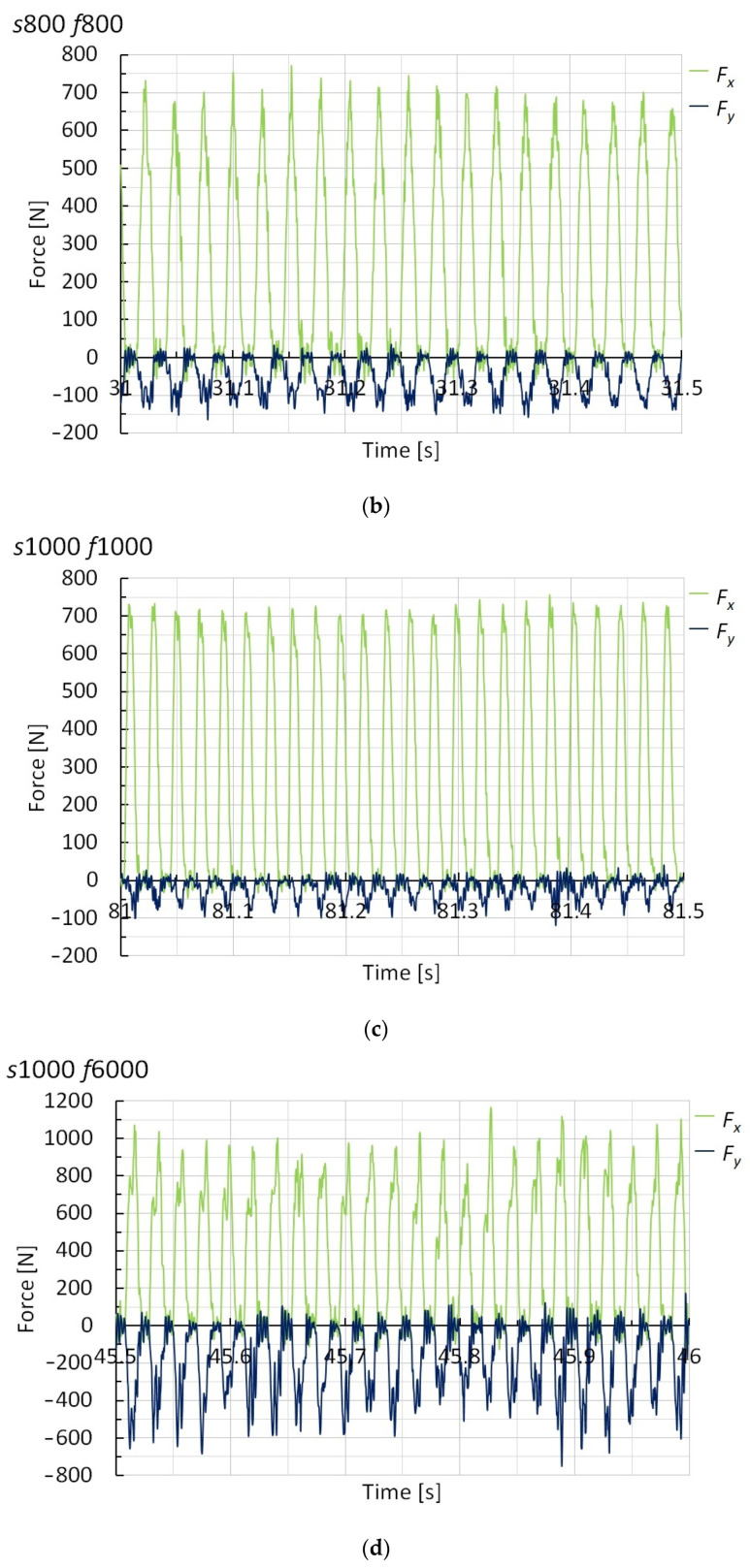
Trend of the cutting forces vs. time for (**a**) *s* = 500 rpm and *f* = 500 mm/min, (**b**) *s* = 800 rpm and *f* = 800 mm/min, (**c**) *s* = 1000 rpm and *f* = 1000 mm/min, and (**d**) *s* = 1000 rpm and *f* = 6000 mm/min.

**Figure 5:**
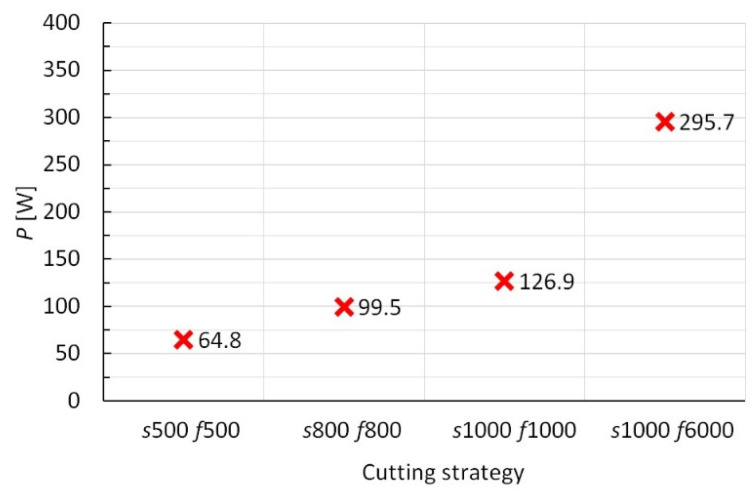
Cutting power values vs. cutting strategies.

**Figure 6:**
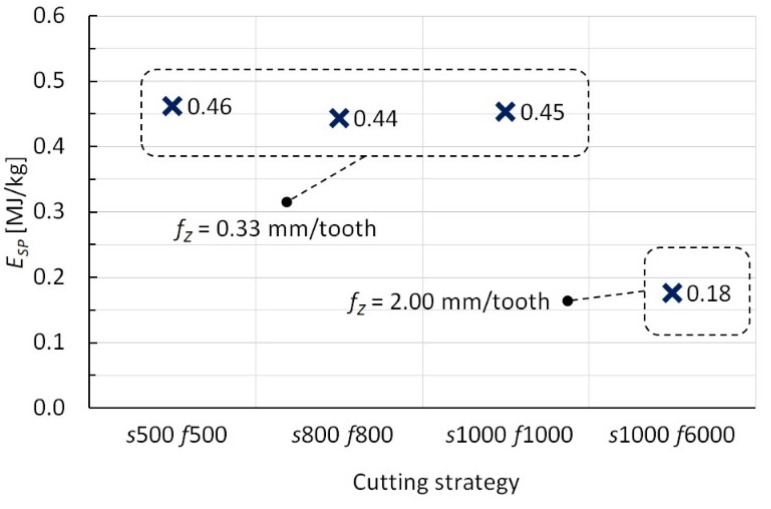
Specific cutting energy values vs. cutting strategies.

Figure 4 reports the cutting forces, *F_x_* and *F_y_*, for the four cutting strategies investigated and for a time interval during which their trend has stabilized. From the mean values of the forces, *P* and *E_SP_* were measured by Equations (1) and (2), respectively. The corresponding values, depending on the cutting strategy, are reported in Figure 5 and Figure 6, respectively.

**Figure 7:**
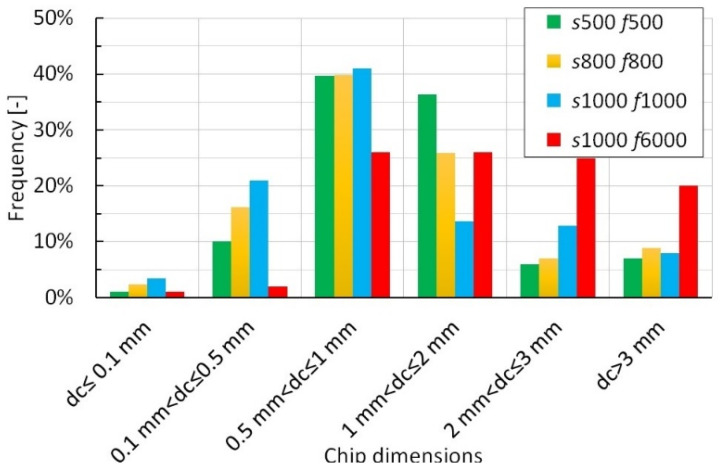
Distribution of the chip dimensions by varying the cutting strategy.

The bar histogram in Figure 7 shows the distribution of the separated chips by sieving as a function of the cutting strategies.

**Figure 8:**
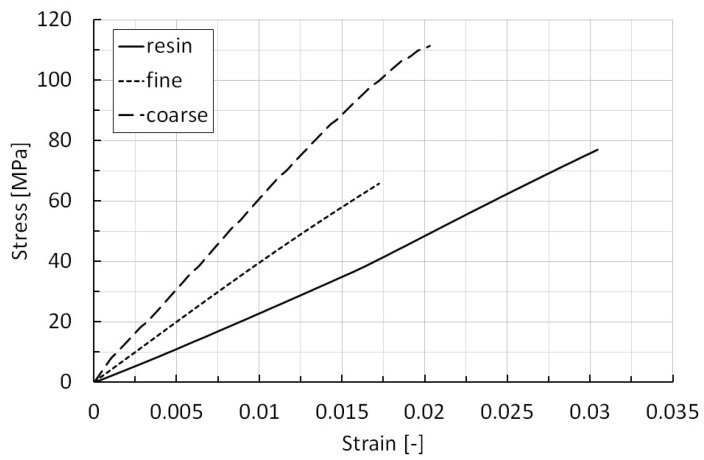
Stress–strain curves from the bending tests on different types of specimens.

Finally, the rectangular specimens were subjected to bending tests; they were made of only resin, resin filled with chips of dimensions *d_c_* > 0.3 mm and *d_c_* ≤ 0.3 mm. Figure 8 reports the exemplary stress–strain curves useful for interpreting the bending behavior of the abovementioned specimens and evaluating the ability of the polymer reinforcing of the machined CFRP, as well as the interfacial adhesion efficiency between the surface of the recovered fibers and the new epoxy matrix; the three sample types are labelled as, in order, resin, coarse, and fine.

## 4. Discussion

From Figure 4, it is possible to note that the cutting forces show a trend reflecting a non-continuous contact of the teeth with the laminates; in some tracts, the forces go to zero, due to their reduced thickness. Moreover, the figure shows that *F_y_* is negative, then opposite to the representation of Figure 1a. Consequently, and about the evaluation of *P*, its contribution to *P_trans_* is negative (although of negligible amount), while it is positive (so as *F_x_*) for the determination of the torque in *P_rot_*.

From Figure 5, it is possible to note that the cutting power, *P*, increases with the cutting speeds; nevertheless, the specific cutting energy is lower for the fastest one, while the first three strategies present similar *E_SP_* values (see Figure 6). Therefore, under the same *f_Z_* value, the cutting speeds do not influence *E_SP_*, while the best result (the lowest *E_SP_* value) is reached by the fourth strategy and is due to more significant crack propagation and/or delamination of the composite layers, because of the higher *f_Z_* value. It is worth reporting that, as stated above, down-milling is an energy-saving strategy compared to up-milling; by way of example, from preliminary tests under the same cutting strategy (*s* = 1000 rpm and *f* = 1000 mm/min), up-milling recorded a value of *E_SP_* equal to 0.80 MJ/kg (almost twice the corresponding down-milling value). Finally, the mechanical process (regardless of the cutting strategy) shows economic and environmental benefits, compared to the embodied energy of virgin CF (183–286 MJ/kg) [33]; concerning this, note that the production of CF is a very energy-intensive process and, consequently, CFs possess higher embodied energies, compared to other synthetic fibers, such as glass ones (about 10 times) [34]. However, it is worth noting that the approach used in this work for the energy evaluation, that is through the cutting forces measurement, does not include the no-load consumption of the milling machine and then furnishes a conservative estimate.

Figure 7 highlights that the dimensions of the chips are strongly influenced by the investigated process parameters. From the figure, it is possible to note that the strategies with the same *f_Z_* value (*s*500 *f*500, *s*800 *f*800, and *s*1000 *f*1000), the amount of fine chips prevails and increases with the cutting speeds; this is due to the fragmentation of the chips generated by the impact with the face of the tool. The amount of coarse chips is higher for the *s*1000 *f*6000 test, then increasing *f_Z_*; this is because the thickness of the chip increases with the feed rate. Generally, the dimensions of the chips are higher than the theoretically calculated ones; this is a consequence of the mechanisms of interlayer delamination and interyarn cracking.

Finally, the bending curves in Figure 8 highlight that each sample is characterized by an almost elastic response with a linear increase in the bending stress, because of the applied deformation. This behavior is strongly evident in both the resin and fine samples; meanwhile, the coarse one shows a slight deviation from the linear behavior with a load decreasing when a stress of about 110 MPa is reached. This change in the bending behavior corresponds to crack formation and damage propagation. All the cases examined show brittle behavior, with the failure occurring for low values of strain and after the nearly linear part of the stress–strain curves. Moreover, from detailed insight into the bending curves, it is possible to conclude that the reinforced samples are characterized by a reduced strain, compared to the resin one. The coarse and fine samples reveal a reduction in the strain at a failure equal to about 32 and 45%. This difference can be attributed to the presence of a longer reinforcement and phenomena of fiber pull-out in correspondence with the failure of the specimens. A focus on the bending curve of the coarse specimens confirms this hypothesis since the deviation from the linear behavior starts almost in correspondence with the maximum strain of the fine specimens. If, on one hand, both the bending curves of the reinforced specimens reveal an improvement in the bending modulus (stress-to-strain ratio; respectively, an increase of about 161 and 80% compared to the resin one, which presents a modulus equal to about 2.2 GPa), and then a stiffer behavior thanks to the introduction of the fibrous reinforcement, on the other hand, the fine one shows a slight reduction in the bending strength of about 14.7%. This last sentence can be attributed to the presence of the recycled CFs in the form of particles in the resin system that, in conjunction with the presence of physical porosities, act as initiation points of internal cracks which nucleate and lead to the premature failure of the specimens. The best results can be attributed to the coarse sample; it shows an improvement in the bending strength of almost 45% in comparison with the resin one (about 77 MPa).

**Figure 9:**
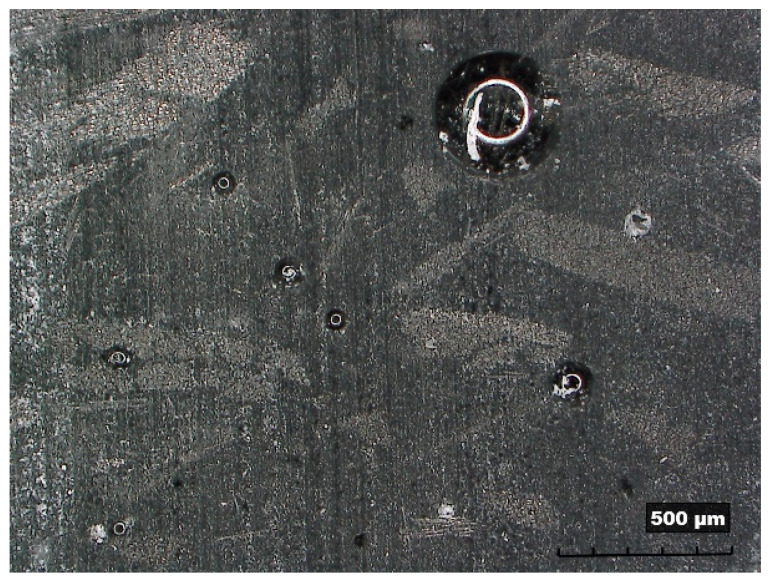
Cross-section of a reinforced specimen with residual porosities.

However, despite all the expedients employed during the production of the specimens, some residual porosities were detected in the recycled material; they can be observed in the proximity of fiber agglomerations, so as highlighted in the cross-section of a reinforced specimen (see Figure 9). This suggests investigating different manufacturing methods for the manufacture of chip-based composite materials.

Finally, it is possible to conclude that, even if it is well known from the literature that the mechanically recycled fibers are characterized by the residues of the matrix on their surface and then by a reduced adhesion efficiency between the recovered fibers and the new resin, the introduction of the fiber-rich recycled reinforcement leads to an improvement in the overall mechanical properties if compared with the pure resin reference. This typology of recycled composite material, although possessing good mechanical properties, cannot be employed in structural applications where high performance is required due to the still poor adhesion at the interface and the reduced aspect ratio.

## 5. Conclusions

This research work follows a machining approach based on peripheral milling for the recycling, in one only step, of CFRP components after the end of their life. The main results highlight that the peripheral down-milling process can represent a viable way for reaching economic and environmental benefits, through the employment of cost-effectiveness cutting tools and an opportune choice of the cutting parameters. In particular, the lowest specific cutting energy, equal to 0.18 MJ/kg, is obtained for the highest value of feed per tooth, equal to 2.00 mm/tooth and corresponding to values of the feed rate and spindle speed of the cutting tool equal to 6000 mm/min and 1000 rpm; on the other hand, the three strategies under the same feed per tooth of 0.33 mm/tooth experience a specific cutting energy of about 0.45 MJ/kg, regardless of the cutting speed values (500 mm/min and 500 rpm, 800 mm/min and 800 rpm, and 1000 mm/min and 1000 rpm). In addition, the chips from the milling process, in particular the ones with dimensions greater than 0.3 mm (these last obtained principally by adopting the strategy with the highest value of feed per tooth), act as an efficient reinforcement for epoxy resins, guaranteeing an increase in stiffness and strength under the bending load; in detail, these features change from 2.2 to 5.8 GPa and from 77 to 111 MPa, respectively.

Future works can consider a deeper investigation of this recycling strategy through, for example, an extended experimental campaign considering further values of the process parameters considered for extending the analysis to other parameters. Moreover, a more accurate chip separation can be considered, as well as the morphological investigation of the chips. Finally, different manufacturing methods of the chip-based composites can be examined, and their mechanical behavior can be extended to several load conditions.

## Data Availability

Not applicable.
